# *Bacillus* sp. alone or combined with salicylic acid inhibited *Trichoderma* spp. infection on harvested white *Hypsizygus marmoreus*

**DOI:** 10.3389/fmicb.2024.1324833

**Published:** 2024-03-18

**Authors:** Xiuqing Yang, Tianhao Li, Yu Liu, Yuyi Gu, Jing Li, Chaoping Wang, Longgang Zhao, Xiaofeng Wang, Wenxiang Li, Yanan Sun, Fansheng Cheng, Dan Zhu

**Affiliations:** ^1^College of Life Science, Qingdao Agricultural University, Qingdao, China; ^2^College of Food Science and Engineering, Qingdao Agricultural University, Qingdao, China; ^3^College of Grassland Science, Qingdao Agricultural University, Qingdao, China; ^4^Shandong Province Key Laboratory of Applied Mycology, Qingdao, China; ^5^Shandong Technology Innovation Center of Special Food, Qingdao Special Food Research Institute, Qingdao, China; ^6^Shandong Academy of Grape, Jinan, China

**Keywords:** postharvest diseases, *Bacillus* sp., *Hypsizygus marmoreus*, antifungal activity, salicylic acid

## Abstract

**Introduction:**

White *Hypsizygus marmoreus* is a popular edible mushroom. It is rich in nutrition and flavor but vulnerable to fungal disease, resulting in nutrient loss and aging.

**Methods:**

In this study, the pathogenic fungus *Trichoderma* spp. BBP-6 and its antagonist *Bacillus* sp. 1–23 were isolated and identified. The negative effects caused by this pathogen were judged by detecting a series of changes in the infected white *H. marmoreus*. The effects of *Bacillus* sp. 1–23 on *Trichoderma* spp. BBP-6 and the infected white *H. marmoreus* were detected. The effect of *Bacillus* sp. 1–23 treatment combined with salicylic acid (SA) was also considered.

**Results:**

The results showed that *Trichoderma* spp. BBP-6 could affect the activities of antioxidant enzymes PAL, POD, CAT, SOD, GR, PPO, and APX to interfere with the stability of the white *H. marmoreus* antioxidant enzyme system and cause the mushroom severe browning and nutrition loss, as well as general quality deterioration. *Bacillus* sp. 1–23 could produce chitinase and chitosanase enzymes to inhibit *Trichoderma* spp. BBP-6 directly. SA reinforced this inhibitory. *Bacillus* sp. 1–23 alone or combined with SA could help white *H. marmoreus* from the *Trichoderma* spp. BBP-6 infection to effectively maintain nutrients, restore and stabilize the antioxidant system, and reduce the production of malondialdehyde, superoxide anion and hydrogen peroxide.

**Discussion:**

Thus, such treatments could be considered potential methods to alleviate damage from disease and extend the shelf life of white *H. marmoreus*.

## 1 Introduction

Mushrooms contain a variety of biologically active ingredients that are beneficial to health, including polysaccharides, vitamins, gamma-aminobutyric acid, ergothioneine and minerals, and hence have formed an integral aspect of the human diet for centuries, both as food and as traditional medicinal supplements (Hu et al., 2021). Other compounds like phenolics (Atila et al., 2023), flavonoids and beta-carotene (Mostafa et al., 2023), amino acids (Sassine et al., 2021), and fatty acids (Abou Fayssal et al., 2021, 2023) found in mushrooms play major roles in the improvement of antioxidant and cardiovascular properties. Moreover, their global consumption has grown significantly in recent years. The commercially available white jade mushroom *H. marmoreus* is particularly popular due to its mildly sweet and nutty flavor, its crunchy texture, and its high levels of potassium, iron, proteins, riboflavin, niacin, vitamin D and other physiologically beneficial constituents (Hu et al., 2020). These substances benefit human health, such as lowering cholesterol content, blood sugar and blood pressure (Yan et al., 2020). Usually, white *H. marmoreus* is consumed fresh since its high respiration intensity, fragile texture and high water content make it susceptible to pathogenic bacteria and fungi infections, which lead to significant browning and spoilage shortly after its harvest (Zeng et al., 2022). The challenging preservation of white *H. marmoreus* has, thus, become a research hotspot in recent years.

Previous research on postharvest mushroom pathogens has focused mainly on bacteria such as *Staphylococcus epidermidis*, *Enterobacter amnigenus*, *Bacillus cereus* and *Pseudomonas* spp. (Carmona-Hernandez et al., 2019) since these bacteria are known to result in significant yield loss, the softening of tissues, and the reduction of shelf-life. For example, *Pseudomonas tolaasii* causes brown blotch disease (Yang et al., 2022), while *Lactococcus lactis* can cause water-soaked and sunken lesions (Zhao et al., 2013). Managing and inhibiting fungal pathogens is particularly challenging since it is difficult to discriminate between pathogenic mycelia and mushroom mycelia during the cultivation stage. *Trichoderma* spp. are a group of widespread pathogenic fungi that cause green mold disease on mushrooms such as *Lentinula edodes* and *Hymenopellis raphanipes* (Wang et al., 2016; Zeng et al., 2022). Since most *Trichoderma* spp. isolates share similar optimal growth conditions to those of mushrooms, it is infeasible to adjust pH value or temperature to control green mold disease (Wang et al., 2022). The release of this kind of pathogen during mushroom transportation and storage, as well as its effects on long-term mushroom quality, are uncertain (Turgay et al., 2023). Traditionally, chemicals like prochloraz-manganese chloride complex and propiconazole were considered effective in inhibiting the pathogen and controlling postharvest disease in mushrooms, however, exposure to these chemicals raises toxicological concerns for humans, animals, and the environment (Zeng et al., 2022). Their safety issues necessitate careful consideration during mushroom cultivation and consumption. Therefore, new and effective methods are urgently developed to prevent and control mold pollution in the harvested mushroom industry.

In plants, salicylic acid (SA) is the main endogenous signaling hormone that promotes biotic stress gene expression (Reymond and Farmer, 1998). It is essential for basal resistance against pathogens, as well as for optimal functioning of the inducible defense mechanism, systemic acquired resistance (SAR), which confers protection against a broad spectrum of pathogens, including those of viral, bacterial, oomycete or fungal origin (Kumar, 2014). SA is recognized as a resistance inducer since it plays an important role in signal transduction during fungal infection, inducing the expression of pathogen-resistance-related genes and acting as an antioxidant to scavenge reactive oxygen species (ROS) (Wani et al., 2017). It also synthesizes defensive compounds, endowing the host with both local and SAR to pathogens (Wani et al., 2017). SA is also known to restrict the growth of necrotrophic pathogen *Alternaria solani* and early blight symptom development in both potato foliage and tubers (Brouwer et al., 2020). Nevertheless, the efficacy of SA against fungal diseases caused by pathogens in mushrooms is unknown.

The postharvest application of microbial antagonists has been introduced as an appropriate and practical method to control disease in harvested fruits and vegetables. *Bacillus* spp. is considered an eco-friendly and bio-safe alternative to traditional chemical fungicides/bactericides due to their intrinsic ability to induce native anti-stress pathways in plants (Lastochkina et al., 2019). Importantly, *Bacillus* sp. are generally recognized as safe microorganisms by the United States Food and Drug Administration (FDA) to be widely applied in the food and drug industry, including the production of enzymes, flavor enhancers, sweeteners, animal feed additives and vitamins (Lastochkina et al., 2019; Zhang et al., 2019). However, to our knowledge, no studies have been conducted using these bacteria to control fungal diseases in white *H. marmoreus*. In this study, the fungal pathogen and the antagonistic bacteria were identified and isolated in white *H. marmoreus*; the effects of the fungal pathogen on postharvest quality were assayed; and the impact of the antagonistic bacteria and SA on postharvest fungal disease and storage quality were evaluated.

## 2 Materials and methods

### 2.1 Isolation and molecular identification of the fungal pathogen and antagonistic bacteria on mushroom

Sub-fresh white *H. marmoreus* was purchased at a local supermarket and transported directly to the laboratory, where disease spot tissues were separated and incubated on potato dextrose agar (PDA) plates. After incubation for 3 d at 28°C, the isolates were re-streaked on new agar plates to isolate the pure cultures for further experiments. Genomic DNA was prepared using a genomic DNA preparation kit (Tiangen, Beijing, China), and the morphological characteristics of the resulting strains were detected under microscopy. Thereafter, the fungal pathogen was re-inoculated and re-isolated from the diseased area to establish Koch’s postulates (Wang et al., 2016). To screen the antagonistic bacteria, 1 g of a soil sample collected from a mushroom cultivation house (in Jiaozhou City, Qingdao, China) was dissolved in 10 mL sterilized saline solution, then serially diluted, inoculated on beef extract-peptone medium plates, and incubated at 37 ± 2°C for 24  h. Colonies were subsequently selected from these plates, and their antagonistic abilities were tested against the pathogens according to the diameters of the fungal growth inhibition zone around the bacteria (Shen et al., 2020).

The bacteria were identified via 16S rRNA gene sequencing using universal primers 27\u00B0F and 1,492 R, while the fungal strain was identified based on internal transcribed spacer (ITS) sequencing using the primers of ITS1 and ITS4. The PCR products were sequenced at Qingdao Weilai Biotechnology Co., Ltd., and the basic local alignment search tool (BLAST) was applied against the GenBank database. A phylogenetic tree was generated using Mega 5.0, in which the screened fungal pathogen and the antagonistic bacteria were named *Trichoderma* spp. BBP-6 and *Bacillus* sp. 1–23, respectively.

### 2.2 Morphological and biochemical characteristics and bacterial chitinolytic enzymes profile analysis

Morphological characteristics of the selected bacterial strains were studied, including colony morphology (color, shape, margin, elevation and surface) and cell morphology (shape, gram reaction and arrangement), and the selected bacteria were subjected to indole, methyl red, Voges-Proskauer, citrate utilization, oxidase and catalase tests, according to standard protocols (Lu et al., 2018). Chitinolytic enzyme profiles were assayed by inoculating the strains onto chitin and chitosan plates, then culturing them at 35°C for 2 d, from which the appearance of the decolorized circle around each strain indicated enzymatic activity.

### 2.3 *In vitro* inhibition of fungal pathogen by SA and *Bacillus* sp. 1–23

Before the bioassay tests, *Trichoderma* spp. BBP-6 was grown on PDA plates at 28°C for 4 d. The bacterial strains *Bacillus* sp. 1–23 were inoculated in a liquid medium of Luria-Bertani (LB) at 37°C, and the cells were harvested at an optical density of OD 600 0.8. The antifungal effect was determined by placing each 6-mm diameter fungal colony in the center of a PDA plate, with 1 μL bacterial suspension or concentration gradient of the SA solution inoculated around it. All the plates were then placed in a 25°C incubator for 5–7 d culture, after which the mycelia growth of the *Trichoderma* spp. BBP-6 was measured to assess the inhibitory effects (Shen et al., 2020).

Subsequently, the *Trichoderma* spp. BBP-6 samples were individually cultured in Czapek Dox liquid medium for 3 d, followed by inoculation with either the *Bacillus* sp. 1–23 or SA at final concentrations of 1 × 10^6^ CFU mL^−1^ and 1 g L^−1^, respectively. After 12 h of co-culturing, the mycelia were harvested by centrifugation and washed twice in sterile double distilled water. The samples were then analyzed using scanning electron microscopy (SEM), according to the previously described method (Swain et al., 2008).

### 2.4 Preparation of fungus pathogen and treatments of mushrooms

The *Trichoderma* spp. BBP-6 was cultured in PDA for 7 d at 25°C, whereafter, the spores were harvested using a glass spreading rod in double distilled water. The spore concentration was adjusted to a final concentration of 1 × 10^6^ spores mL^−1^ using a hemocytometer (Li et al., 2019).

Fresh white *H. marmoreus* H8 were harvested from Shandong Fanghua Mushroom Co., Ltd. (Shandong, China), and then transported immediately to the laboratory in a foam box, ensuring a constant temperature to minimize physical damage and temperature fluctuation. Mushroom bodies of uniform size and maturity, and with no sign of disease or damage, were selected for this study and were randomly divided into four groups. Each group contained three replicates, and each replicates weighed 1 kg. The CK group was treated with distilled water for 2 min and then air-dried, while the T group was treated with *Trichoderma* spp. BBP-6 (1 × 10^6^ spores mL^−1^) in the same way. Samples in the B group and B + S group were treated with *Bacillus* sp. 1–23 (1 × 10^8^ CFU mL^−1^) or *Bacillus* sp. 1–23 (1 × 10^8^ CFU mL^−1^) +3 g/L SA for 30 s, respectively, then air-dried and sprayed with a conidial suspension of *Trichoderma* spp. BBP-6 at 10^6^ spores mL^−1^ (Essghaier et al., 2009). All the samples were then placed on plastic trays and stored at 22°C and 85–90% relative humidity for 6 d. Mushroom stipes of 1–4 cm below the cap were sampled each day, then cut into small pieces, frozen in liquid nitrogen, and stored at −80°C for further analysis.

### 2.5 Determination of weight loss ratio, browning degree, reducing sugar and soluble protein content

According to a previous report, weight loss was measured, recorded, and expressed as the percentage of weight loss relative to the initial weight (Zhu et al., 2021). For protein and reducing sugar extraction, 3 g of frozen mushrooms was homogenized with 6 mL of PBS buffer (50 mM, pH 7.8). The homogenized mixture was centrifuged at 10,000 g for 20 min at 4°C, and the reducing sugar content and total soluble protein were determined according to both the 3,5-dinitrosalicylic acid (DNS) and Bradford methods, using glucose and bovine serum albumin, respectively, as standards, expressed as g kg^−1^ (Wang et al., 2021).

### 2.6 Determination of malondialdehyde (MDA), superoxide anions and hydrogen peroxide content

As an indicator of the degree of lipid peroxidation, malondialdehyde (MDA) levels in the groups were determined as previously reported, with some modification (Zhu et al., 2019). Each 0.5 g sample was homogenized in 2 mL trichloroacetic acid (TCA, 5% w/v). After centrifugation for 10 min at under 4°C, 1 mL of the supernatant was added to 1 mL 2-thiobarbituric acid (0.6% w/v), then incubated in boiling water for 20 min. After cooling, the supernatant of the reaction mixture was determined at three different wavelengths (450, 532 and 600 nm), and MDA content was calculated accordingly, expressed as mmol kg^−1^.

For the determination of superoxide anions, 1 g of white *H. marmoreus* sample was homogenized in 6 mL PBS in an ice bath, and the supernatant was obtained via centrifugation at 4°C. The reaction mixture contained 1 mL supernatant solution, 1 mL 0.01 M hydroxylamine hydrochloride, 1 mL 20 mM p-aminobenzene sulfonic acid and 1 mL 0.05 M α-naphthylamine, with potassium nitrite employed as the standard. After being left to react at 25°C for 10 min, the absorbance was determined at 530 nm (Hu et al., 2020), expressed as mmol min^−1^ kg^−1^.

For hydrogen peroxide (H_2_O_2_), 0.5 g of sample tissue was homogenized with 4 mL of TCA (0.1% w/v) under ice conditions, whereafter the homogenate was centrifuged, and 0.5 mL supernatant was mixed with 0.5 mL potassium phosphate buffer (10 mM, pH 7.0) and 1 mL 10 M KI. The absorbance was subsequently recorded at 390 nm, expressed as mmol kg^−1^ (Khan et al., 2014).

### 2.7 Determination of enzymatic activity

A total of 3 g sample was ground into a homogenate with 6 mL of potassium phosphate buffer (pH 7.8) under ice-cold conditions. The supernatant of the homogenate was obtained after centrifugation at 10,000 × g for 20 min at 4°C as the crude enzyme solution. According to a previous report, the cellulase (EC 3.2.1.4) activity was determined using glucose as the standard (Zhu et al., 2019). The cellulase activity is defined as the amount of glucose (U) ultimately produced per kilogram of cellulase per minute at 37°C (unit: U kg ^− 1^). In addition, for polyphenol oxidase (PPO, EC 1.10.3.2) determination, 0.4 mL of catechol and 2.5 mL of acetic acid buffer were bathed at 25°C for 10 min, during which OD_410_ was recorded every 30 s via the addition of 0.5 mL crude enzyme solution. PPO activity was expressed in U kg^−1^ (Liu et al., 2019).

To analyze the phenylalanine ammonia-lyase (PAL, EC 4.3.1.5) activity, 200 μL crude enzyme solution was incubated with 2 mL borate buffer (50 mM, pH 8.8) and 1 mL 20 mM *L*-phenylalanine at 30°C for 30 min. The reaction was terminated using 1 μL 6 M HCl. PAL activity was determined according to the production of cinnamate, which was measured at an absorbance of 290 nm. One unit of PAL enzymatic activity was defined as an increment of 0.01 at 290 nm per kilogram of protein and expressed as U kg^−1^ (Zhu et al., 2019).

The guaiacol method was used to determine peroxidase (POD, EC1.11.1.7) activity (Wang et al., 2005), with 0.5 mL of crude enzyme solution incubated in 2 mL substrate buffer (100 mM sodium phosphate, pH 6.4 and 8 mM guaiacol) for 5 min at 30°C. The increasing absorbance was determined at 460 nm for 120 s after adding 1 mL of 24 mM H_2_O_2_. One unit of POD activity was defined as an increase of 0.1 in absorbance per minute at 470 nm per gram of protein and expressed as U kg^−1^.

To determine the catalase (CAT, EC 1.11.1.6) activity, 100 μL crude enzyme solution was added to 3 mL 20 mM H_2_O_2_. A spectrometer was used to determine the absorbance at 240 nm for 2 min. A unit of CAT activity was defined as the amount of enzyme required to decompose 0.1 M H_2_O_2_ per minute at 30°C (Wang et al., 2005), expressed as U kg^−1^.

The superoxide dismutase (SOD EC 1.15.1.1) activity assay was conducted according to a previous study, with slight modifications (Liu et al., 2019). A 100 μL crude enzyme solution was added to a reaction solution containing 13 mM methionine, 25 mM nitroblue tetrazolium chloride (NBT), 0.1 mM ethylenediamine tetraacetic acid (EDTA), 50 mM phosphate buffer (pH 7.8), and 50 mM sodium carbonate. The reaction began with adding 2 μM riboflavin, after which the tubes were placed under two 4,000 lux daylight lamps for 15 min. The control was a complete reaction mixture devoid of any enzyme. The reaction was stopped by switching off the light and placing the tubes into darkness. A non-irradiated complete reaction mixture served as a blank. SOD activity was estimated according to the recorded decrease in the optical density of the NBT dye caused by the enzyme. The absorbance was recorded at 560 nm, and one unit of SOD activity was defined as the amount of enzyme that had caused a 50% inhibition of NBT, expressed as U kg^−1^.

The determination of ascorbate peroxidase (APX, EC 1.11.1.11) activity was based on the decrease in the absorbance of ascorbic acid at 290 nm. The reaction mixture contained 0.1 mL of crude enzyme solution, 100 mM phosphate buffer (pH 7.0), 5 mM ascorbate, 0.1 mM EDTA, and 1.2 mM H_2_O_2_. The decrease in absorbance at 290 nm was recorded for 3 min and APX activity was expressed as U kg^−1^ (Khan et al., 2014).

The determination of glutathione reductase (GR, EC 1.6.4.2) activity was conducted according to the kit instructions of the manufacturer (Nanjing Jiancheng Bioengineering Institute, China), at pH 8.0, 1 mmol NADPH oxidation per mg of protein per minute was catalyzed as one unit of enzyme activity, expressed as U kg^−1^.

### 2.8 Statistical analysis

All experiments were conducted according to a completely randomized design, and significant differences were determined by one-way analysis of variance (ANOVA), followed by Duncan’s multiple-range comparisons. The data is presented as the mean ± standard deviation, and differences were considered significant at *p* < 0.05. All the figures were created with Origin 9.0 software (Origin Lab, Massachusetts, USA).

## 3 Results

### 3.1 Identification of the fungal pathogens and antagonistic bacteria

**Figure 1:**
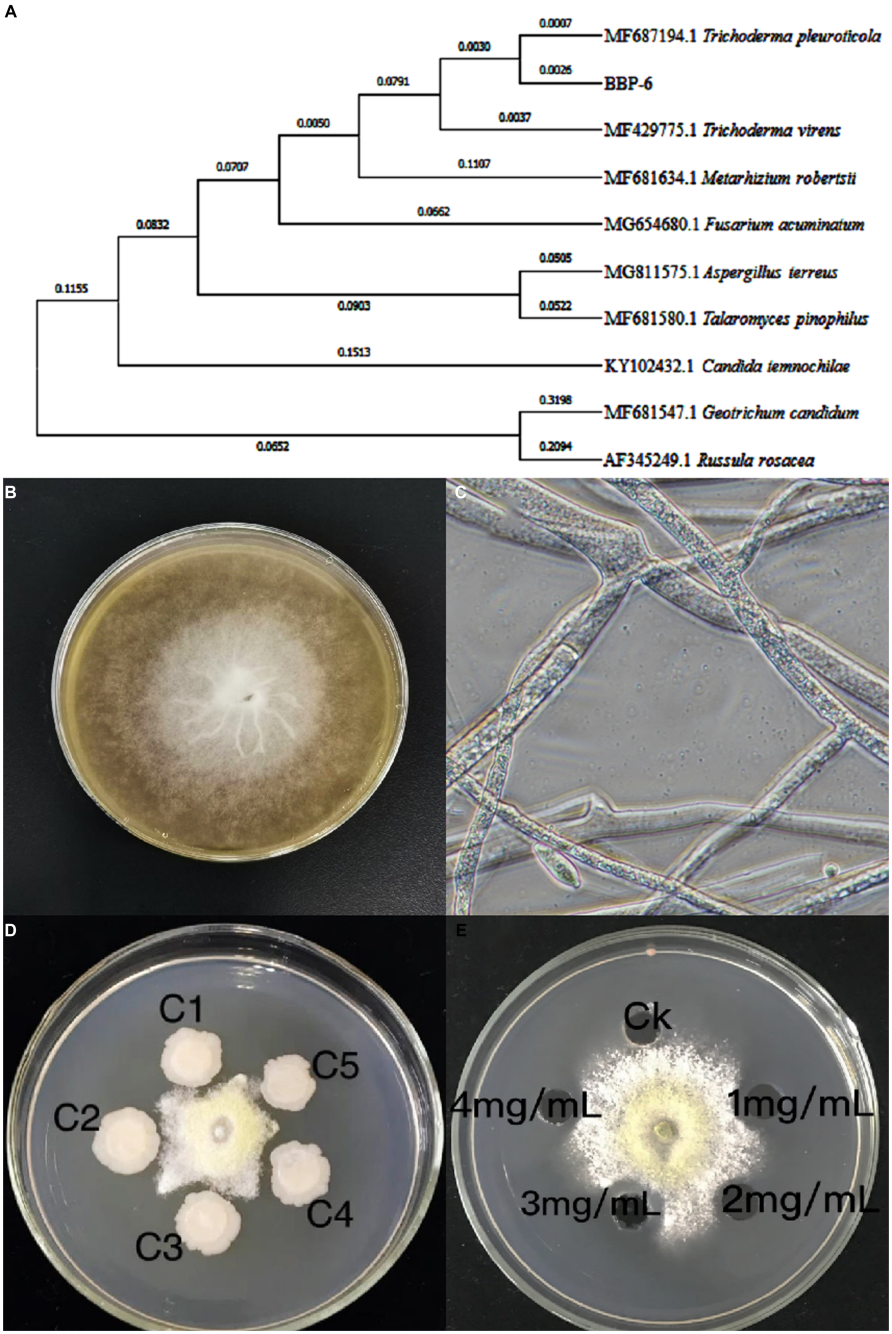
Phylogenetic analysis of fungal pathogens *Trichoderma* spp. BBP-6 **(A)**; The *Trichoderma* spp. BBP-6 mycelium on PDA medium **(B)** and under microscopy **(C)**; Inhibitory effects of *Bacillus* sp. 1–23 **(D)** and salicylic acid **(E)** on *Trichoderma* spp. BBP-6.

In this study, one strain was isolated from a diseased mushroom, which typically occurred on the stipe of the mushroom with white aerial hyphae, and was found to be identical to the mushroom mycelium. The fungal strain was identified by ITS sequencing (Figure 1A) as belonging to *Trichoderma* genus, and most similar to *Trichoderma pleuroticola*.

The fungal colonies first produced floccose and white septate hyphae, after which dark green hypha appeared (Figure 1B). The conidia formed tree-like, right-angle branched conidiophores and were either glaucous, single, ellipsoidal or subglobose, ranging from 2.2 to 3.8 × 2.3 to 3.5 μm. Primary conidiophore branches arose singly or in pairs. Phialides were ampulliform, slightly constricted at the base, which measured 1.3 to 3.2 μm, were swollen in the middle, which measured 6.0 to 8.5 μm, and then narrowed abruptly at the apex (Figure 1C). These features closely matched those of the *Trichoderma* sp., and the strain was accordingly named *Trichoderma* spp. BBP-6.

In addition, soil from the mushroom farming house was collected and screened for the isolation of antagonistic bacteria. One gram-positive strain, featuring a rod shape and spores, was successfully isolated, and phylogenetic analysis via 16S rRNA revealed that this strain belonged to the *Bacillus* genus and was closely related to *B. subtilis*. The strain also tested positive in the catalase, oxidase, citrate utilization and Voges-Proskauer tests, but negative in the urease, methyl red and indole tests. These physiological and biochemical identification results were found to match the obtained 16S rRNA sequencing data as well as the description of *B. subtilis* (Lu et al., 2018).

### 3.2 Inhibitory effects of *Bacillus* sp. 1–23 and SA on *Trichoderma* spp. BBP-6

As indicated in Figure 1D, the inoculation of *Bacillus* sp.1–23 remarkably inhibited the growth of the pathogenic fungus *Trichoderma* spp. BBP-6. Meanwhile, compared with the results of the control group, the SA exhibited antifungal activity against *Trichoderma* spp. BBP-6 (Figure 1E) in a dose-dependent manner, which was most effective at a concentration of 3 g/L.

**Figure 2:**
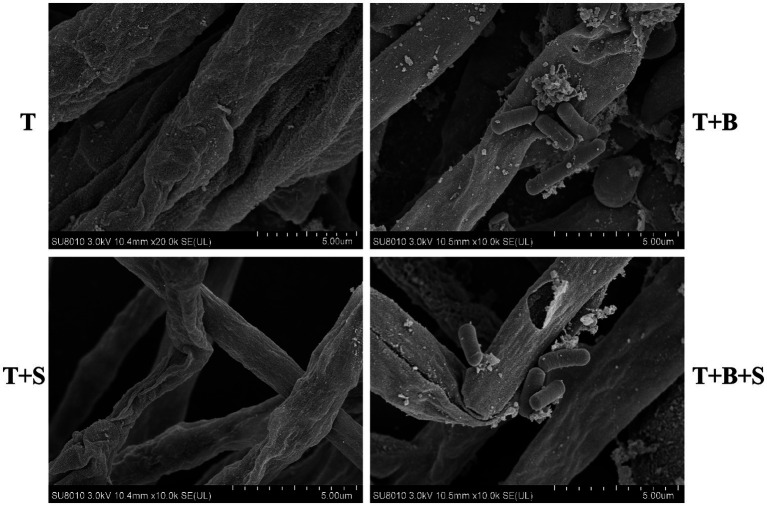
SEM results of different treatments on *Trichoderma* spp. BBP-6. **(A)** T: *Trichoderma* spp. BBP-6; **(B)** T + B: Co-cultured *Trichoderma* spp. BBP-6 and *Bacillus* spp. 1–23; **(C)** T + S: *Trichoderma* spp. BBP-6 treated with SA; **(D)** T + B + S: *Trichoderma* spp. BBP-6 treated with *Bacillus* spp. 1–23 and SA.

SEM imaging in this study indicated the proliferation of *Bacillus* sp. 1–23 cells on the hyphae of *Trichoderma* spp. BBP-6. Moreover, the fungal hyphae of *Trichoderma* spp. BBP-6 (Figure 2A) incubated with *Bacillus* sp. 1–23 demonstrated the combination, interaction, alternation and destruction of the hyphal cell wall (Figure 2B). The strain in this study was further investigated by extracellular enzyme profiling, the results of which indicated its capabilities in terms of chitinase and chitosanase screening (data not shown). SA treatment led to distorted, slimy and swollen hyphae in *Trichoderma* spp. BBP-6, with deficient growth phenotypes (Figure 2C), which suggested that SA induced hyphal cell death, thereby functioning as a chemical fungicide (Li et al., 2022). Moreover, the combined treatment of *Bacillus* sp. 1–23 and SA led to severe cytoplasmic content damage and the evident lysis of *Trichoderma* spp. BBP-6 hyphae cell wall was observed (Figure 2D).

### 3.3 Effects of different treatments on mushroom browning

**Figure 3:**
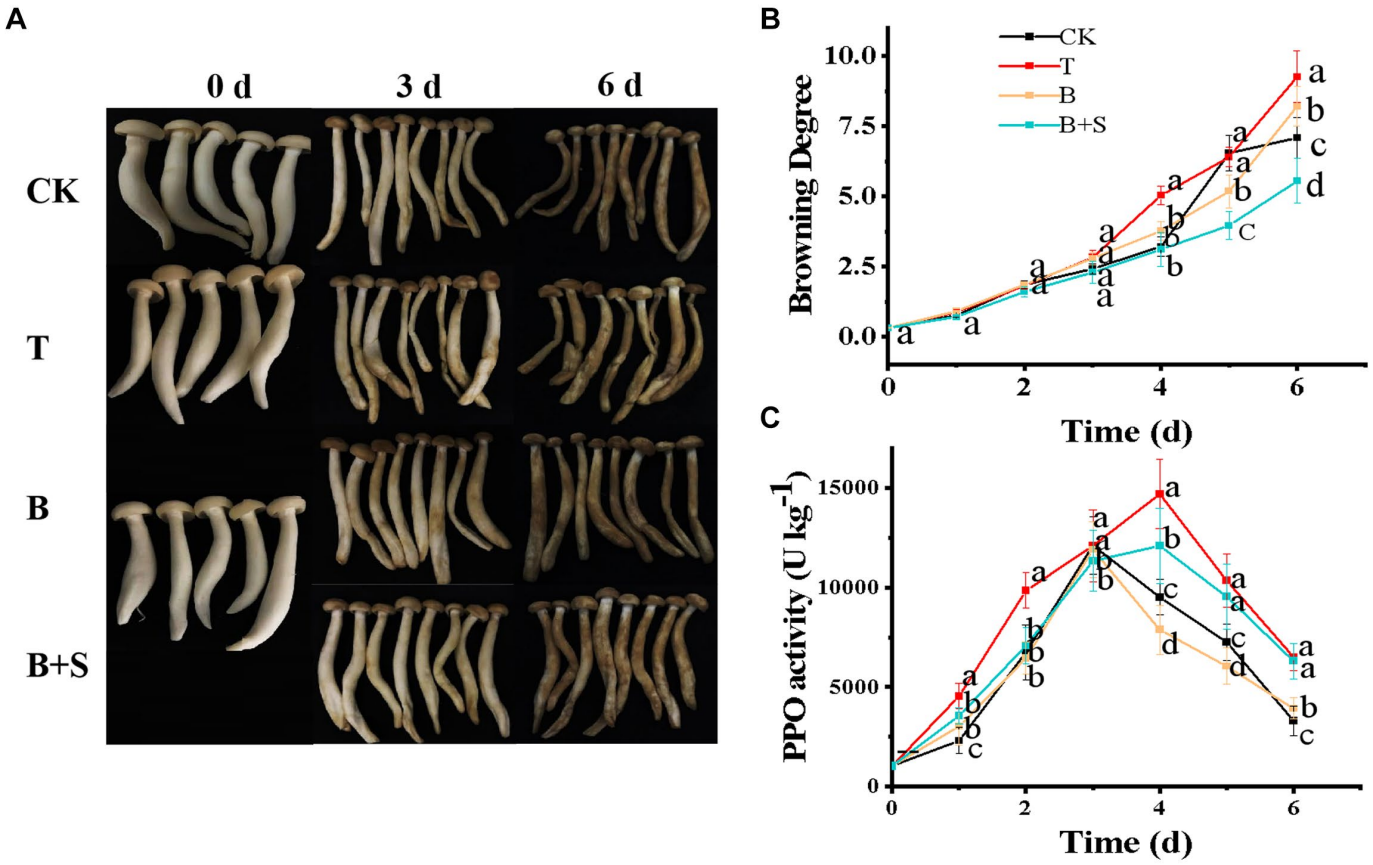
Effects of different treatments on the white *H. marmoreus* appearance **(A)**, browning degree **(B)** and PPO activity **(C)**. CK refers to control; T: white *H. marmoreus* treated by *Trichoderma* spp. BBP-6; B: Infected white *H. marmoreus* treated with *Bacillus* sp. 1–23; B + S: Infected white *H. marmoreus* treated with *Bacillus* sp. 1–23 and SA. Each value is displayed as the mean ± SD (*n* = 3).

Browning is a direct indicator of deterioration and, thus, reflects the freshness of a mushroom. Figure 3A depicts the apparent variations in white *H. marmoreus* following the different treatments in this study. All mushroom samples underwent continuous quality deterioration during the entire duration of their storage, with apparent browning, water loss and texture shrinkage. However, compared with the CK group, the appearance of mushrooms in the T group was worse, but the B and B + S groups were much better. The most severe browning was observed in the T group samples during storage, and the browning degree of the different treatments increased dramatically from day 4 (Figure 3B). Furthermore, this deterioration was accompanied by a fetid odor and water loss, indicating that the *Trichoderma* spp. BBP-6 infection may have promoted bacterial pollution during the later stage of storage (Zhang et al., 2018; Lin and Sun, 2019). Compared with the CK group, *Trichoderma* spp. BBP-6 infection severely promoted the degree of browning. However, this trend was attenuated by the alone or combined treatment of *Bacillus* sp. 1–23 and SA. The results in Figure 3B were in accordance with the results shown in Figure 3A. The PPO is tyrosinase and the key enzyme in the browning process (Lin and Sun, 2019). As illustrated in Figure 3C, all of the groups in this study exhibited a bell-shaped trend. Notably, PPO enzyme activity in the T group remained at a higher level compared with that of other groups, indicating that the *Trichoderma* spp. BBP-6 infection obviously enhanced the PPO activity of white *H. marmoreus*. In contrast, the mushrooms in the B group exhibited the lowest PPO activity of all four groups after 3 d, indicating the ability of the *Bacillus* sp. 1–23 treatment to inhibit the PPO activity of white *H. marmoreus* in later stages of storage.

### 3.4 Effects of different treatments on weight loss ratio, reducing sugar and soluble protein contents

**Figure 4:**
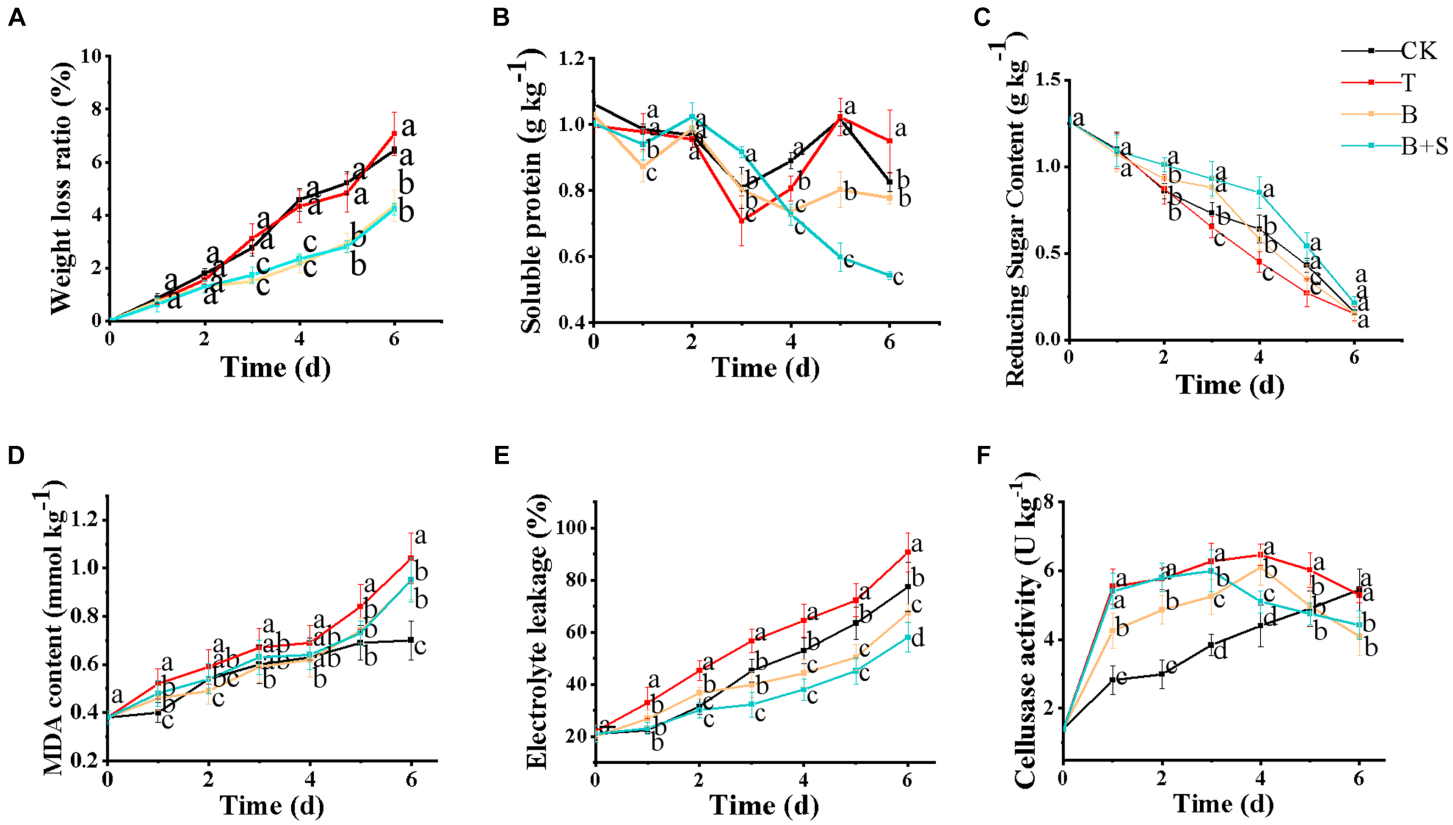
Effects of different treatments on the weight loss ratio **(A)**, soluble protein **(B)**, reducing sugar content **(C)**, MDA **(D)**, electrolyte leakage **(E),** and cellulase activity **(F)** of white *H. marmoreus*. Each value is displayed as the mean ± SD (*n* = 3).

Since mushrooms lack a protective outer shell and are characterized by a high moisture content and respiration rate, severe quality loss can also be attributed to their susceptibility to dehydration (Wang et al., 2021). Here, on day 7, the weight loss ratios of white *H. marmoreus* groups treated with sterile distilled water or *Trichoderma* spp. BBP-6 infections were approximately 8% and were higher than those of either the B or B + S groups (Figure 4A).

Soluble protein and reducing sugar are important nutrient indicators of physiological and biochemical changes, as well as changes in quality during storage (Li et al., 2019). The soluble protein contents in the B and B + S groups were steadily downward (Figure 4B). This result was in accordance with that of the same research on the mushroom *Pholiota nameko* (Zhu et al., 2019). However, the soluble protein contents in the T and CK groups fluctuated at the later stage of storage, which may be caused by pathogenic infection (Zhang et al., 2018). The reduced sugar content of all the differently treated mushrooms showed a decreasing trend with storage time. Notably, the T group and the B + S groups ranked lowest and highest, respectively, in their levels of reducing sugar during storage (Figure 4C).

### 3.5 Effects of different treatments on MDA, electrolyte leakage and cellulase activity

MDA, a byproduct of membrane lipid peroxidation, can directly reflect the extent of membrane damage. A high level of MDA content indicates a high degree of membrane lipid peroxidation (Lin et al., 2017; Duan et al., 2022). Here, the dynamics of MDA content after the different treatments are demonstrated in Figure 4D, in which all groups show an increasing trend. The MDA content of the T group was found to be relatively high compared to that of other groups, demonstrating that the *Trichoderma* spp. BBP-6 infection may have led to severe membrane lipid peroxidation. Comparatively, the MDA contents in the CK, B + S and B groups exhibited no significant differences in the first 5 days of storage.

Additionally, electrolyte leakage, one of the critical biomarkers of membrane unsaturation fatty acids peroxidation, can be used to estimate membrane quality attributes such as fluidity and permeability (Ding et al., 2016). As revealed in Figure 4E, electrolyte leakage in all mushrooms generally increased during storage, consistent with the browning index trend (Dokhanieh Yousefpour and Aghdam Soleimani, 2016). Additionally, the *Trichoderma* spp. BBP-6 infection enhanced electrolyte leakage in the T group compared with the CK group, while the B and B + S groups exhibited lower levels of electrolyte leakage. In a previous study, button mushrooms treated with 250 μM SA exhibited lower electrolyte leakage than untreated mushrooms, which is in line with the findings of the present study (Dokhanieh Yousefpour and Aghdam Soleimani, 2016).

Mushrooms soften in texture via the degradation of the cell wall by endogenous autolysins or microbial infection during storage. In particular, cellulase, comprising a complex of enzymes, including exoglucanase, endoglucanase and β-glucosidase, has been shown to degrade mushroom tissue and decrease its firmness (Zhu et al., 2019). As illustrated in Figure 4F, the CK group exhibited the lowest level of cellulase in the first five days of storage with a continuously increasing trend compared with other groups, which was similar to the same research result observed in *P. nameko* (Zhu et al., 2019). The *Trichoderma* spp. BBP-6 infection induced the enhancement of cellulase activity, while the *Bacillus* sp. 1–23 alone or combined with SA treatments could remarkably alleviate this change.

### 3.6 Effects of different treatments on superoxide anion production rate and hydrogen peroxide content

**Figure 5:**
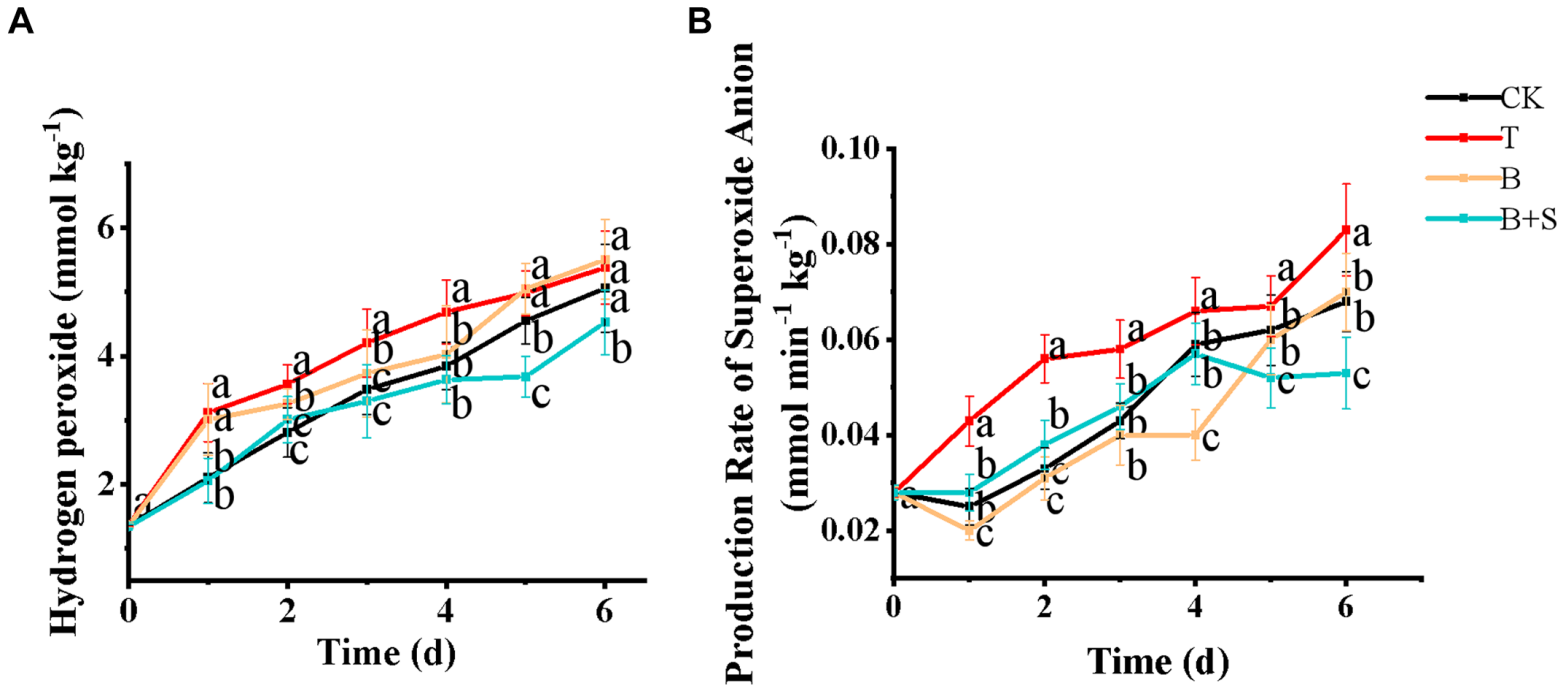
Effects of different treatments on the hydrogen peroxide **(A)** and production rate of superoxide anion **(B)** of white *H. marmoreus*. Each value is displayed as the mean ± SD (*n* = 3).

The production of ROS, such as superoxide anions and H_2_O_2_, is an intrinsic feature of senescence, as high levels of ROS can damage several cellular components, including DNA, proteins and lipids (Liu et al., 2019). A cytotoxic metabolic waste, H_2_O_2_ can penetrate most cell membranes, and higher levels of H_2_O_2_ can be induced by pathogenic infections, such as the pepper gray mold disease attacked by *Botrytis cinerea* (Jiang et al., 2018). As demonstrated in Figure 5A, H_2_O_2_ in the T group remained at a higher level during storage compared with the CK group, while the B and B + S treatments reduced H_2_O_2_ levels, with the lowest ones noted in the *Bacillus* sp. 1–23 and SA combination treatment. Similarly, button mushrooms treated with 250 μM SA exhibited lower H_2_O_2_ accumulation than those treated without SA (Dokhanieh Yousefpour and Aghdam Soleimani, 2016). The T group also exhibited the highest superoxide anion production rate (Figure 5B), while, in contrast, the superoxide anion production rate in mushrooms treated with *Bacillus* sp. 1–23, whether individually or in combination with the SA, obviously decreased.

### 3.7 Effects of different treatments on antioxidant defensive enzymes activity

**Figure 6:**
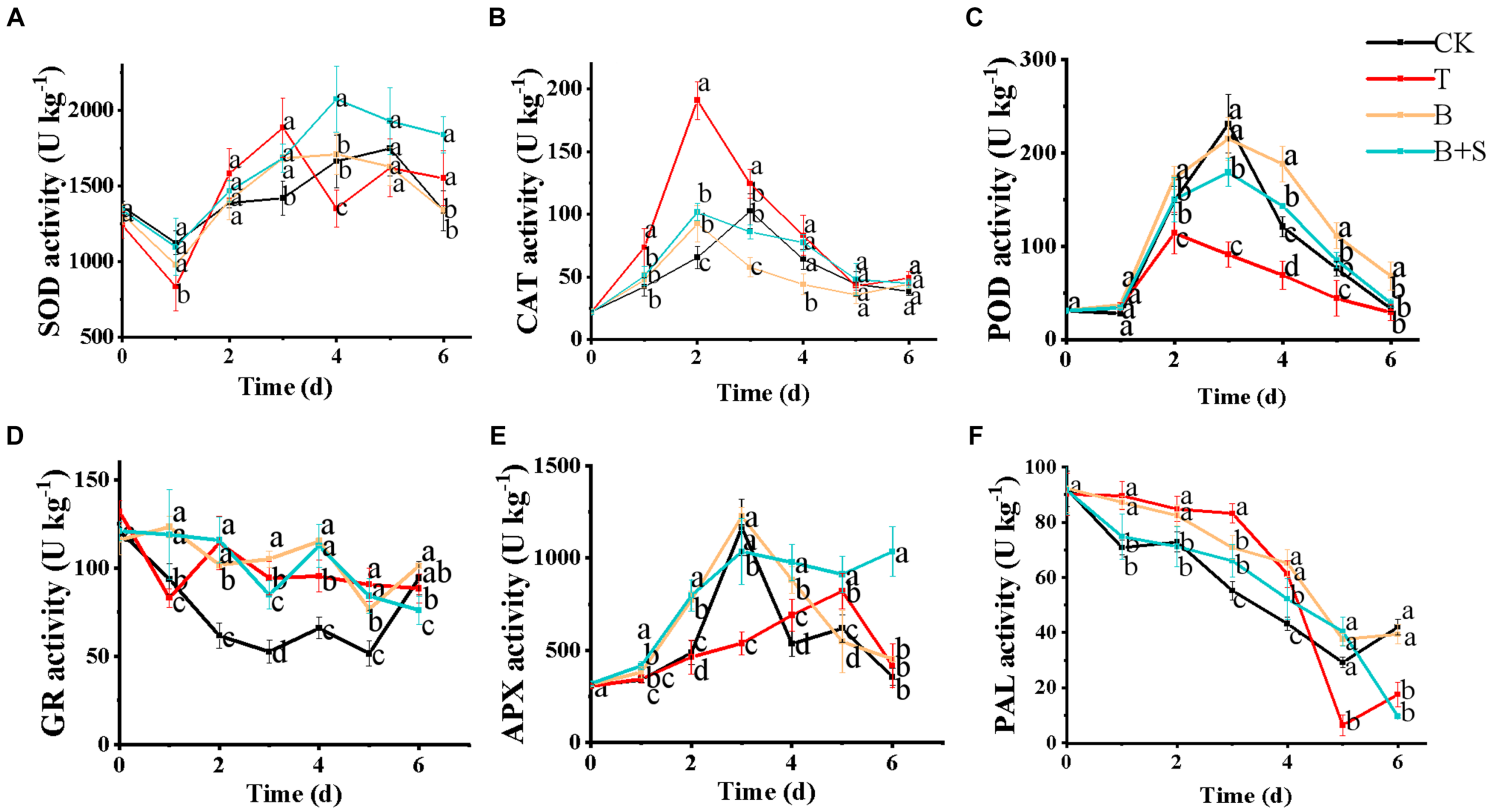
Effects of different treatments on the SOD **(A)**, CAT **(B)**, POD **(C)**, GR **(D)**, APX **(E)**, and PAL **(F)** activities of white *H. marmoreus* during the storage. Each value is displayed as the mean ± SD (*n* = 3).

The oxygen-free radical scavenger SOD can catalyze the dismutation of O^2−^ to produce H_2_O_2_, which is then removed by CAT and POD. Both SOD and CAT protect cells from peroxides and are closely related to stress resistance and defense against aging (Hu et al., 2020). As illustrated in Figure 6A, the SOD enzyme activity of all treatment groups generally showed an upward trend in the first few days, followed by a downward trend. Compared with the CK group, the inoculation of *Trichoderma* spp. BBP-6 on white *H. marmoreus* in the T group induced SOD enzyme activity that peaked on day 3, while the SOD peak in the CK group occurred on day 5. The SOD level in the B + S group was much higher than that in the CK group and it peaked on day 4.

The CAT activities in the different treatment groups are illustrated in Figure 6B. The CAT level in the CK group exhibited a fluctuating trend and peaked on day 3, while the CAT level in the T group was higher than that of the CK group and peaked earlier, on day 2. Both the B and B + S groups showed similar trends to that of the T group but with lower activity levels.

POD, an enzyme with multiple functions, is associated with ROS metabolism, browning, lignin synthesis and disease resistance in fruits (Yan et al., 2019). As revealed in Figure 6C, the POD activity in all four groups exhibited a bell shape, and activity in the CK, B and B + S groups peaked on day 3, while that in the T group peaked on day 2 and was notably lower than the activity in the other groups. The B and B + S groups also exhibited higher levels of POD activity compared with the CK group during the late stage of storage.

GR is a critical enzyme in the glutathione redox cycle and catalyzes the reduction of oxidized glutathione to reduced glutathione (Zhang et al., 2022). Here, in all four groups, GR activity generally exhibited a downward trend but was enhanced by *Trichoderma* spp. BBP-6 pathogen inoculation. GR activities in the B and B + S groups waved during storage and remained higher than the CK group (Figure 6D).

In the assessment of APX activity in white *H. marmoreus* (Figure 6E), all groups showed an overall trend of first increasing and then decreasing. Activity peaked in the T group on day 5, which was 3 days later than the APX peaks observed in the CK, B and B + S groups. Furthermore, APX activity in these three groups was much higher than that in the T group, indicating that the *Bacillus* sp. 1–23 treatment, both individually and in combination with the SA, had relieved the *Trichoderma* spp. BBP-6 infection in the mushrooms.

PAL, an enzyme in the phenylpropanoid pathway, is linked to the biosynthesis of various natural products, such as lignins, pigments, flavonoids and phytoalexins. PAL activity is influenced by biotic and abiotic responses, including pathogenic attacks, mechanical injury and low temperatures (Bill et al., 2017). As demonstrated in Figure 6F, the PAL enzyme activity in all treatment groups gradually decreased. The PAL enzyme activity in the T group was maintained to be higher than that in the CK group during the first 4 d storage. Interestingly, this variation trend was reversed on the 5th d and 6th d. these results showed that the effects of *Trichoderma* spp. BBP-6 infection in PAL activities was mitigated after the treatment of *Bacillus* sp. alone or combined with SA.

## 4 Discussion

Chitin is one of the main components in the fungal pathogen cell wall (Brown et al., 2020). However, due to its direct chitinase activities, the genera *Bacillus* is considered a remarkably efficient antagonist and phytopathogen controller (Carmona-Hernandez et al., 2019). For example, in one study, the chitinase activity of *B. subtilis* inhibited the growth of up to 83% of *Fusarium oxysporum* and *Botryodiplodia theobromae* infections in yam, and the breakdown of fungal cell walls was hypothesized to have been caused by chitinase production (Swain et al., 2008). Furthermore, chitosanase produced from the bacterium *Bacillus cereus* D-11 remarkably inhibited the mycelial growth of *Rhizoctonia solani* on PDA medium, a plant pathogenic fungus with a wide host range and worldwide distribution (Gao et al., 2008; Swain et al., 2008). These results are further substantiated by our evidence of *Bacillus* sp. 1-23’s production of chitinases and chitosanase.

Numerous factors, such as mechanical damage, exposure to phenolic compounds, pathogens, and postharvest environment, can affect the color change of mushrooms. At present, targeted measures, such as the usage of modified atmosphere packaging or coatings, PPO inhibitors, and irradiation technologies, have been developed to reduce this change process (Lin and Sun, 2019). In addition to PPO, POD is also the main enzyme that causes browning (Nasiri et al., 2019), but it is worth noting that *Trichoderma* spp. BBP-6 inhibited POD activity and PPO content in white *H. marmoreus* was much higher than POD content (Figure 6C and Figure 3C), so the browning degree of white *H. marmoreus* was mainly related to PPO activity. These results concurred with those of our study regarding browning index and appearance quality. The results of this research were also consistent with a previous report showing that the SA treatment at 250 μM lowered PPO activity and delayed cap browning of the button mushroom (Dokhanieh Yousefpour and Aghdam Soleimani, 2016). Our novel finding, thus, provides a biological choice to control the browning process.

The activities of various oxidases, such as SOD, CAT, GR, PPO and PAL, were increased to varying degrees in white *H. marmoreus* to respond to the stress of *Trichoderma* spp. BBP-6. These changes might accelerate the excessive consumption of matter and energy in white *H. marmoreus*, resulting in accelerated aging and deterioration. The activity of these enzymes was increased, and POD activity was suppressed, so the antioxidant system of white H. marmoreus was out of balance. *Bacillus* spp. is considered an eco-friendly and bio-safe alternative to traditional chemical fungicides/bactericides due to their intrinsic ability to induce native anti-stress pathways in plants (Lastochkina et al., 2019). This phenomenon was observed after the *B. subtilis* JK-14 treatment on the peaches infected by *Alternaria tenuis* and *B. cinerea*. The activity of antioxidant enzymes SOD, POD and CAT in the diseased peaches was effectively enhanced, and the growth of pathogens was inhibited (Zhang et al., 2019).

It was shown that *Bacillus* could directly destroy the pathogens, so the antioxidant enzyme activity in the treatment groups was restored to the level of the control group. The enzyme activity was less fluctuating but not significantly increased. Therefore, it was more inclined to believe that *Bacillus* did not directly activate the antioxidant system of white *H. marmoreus*. The aging and deterioration of white *H. marmoreus* could be alleviated only by reducing the biological stress. Our findings do not conflict with other previous reports, such as *Bacillus subtilis* was reported to be the most active antagonist among citrus fruit pathogens, and was shown to protect against *Penicillium digitatum*, *Alternaria citri* and *Geotrichum candidum* (Lastochkina et al., 2019). In another study, five endophytic *Bacillus* spp. were screened for antagonistic effects against *Fusarium oxysporum* in tomatoes, using bacterial whole-cell suspensions and cell-free culture filtrates. All these strains could completely suppress the pathogen’s sporulation and mycelial growth due to their ability to induce systemic resistance by producing lipopeptide antibiotics and chitinase (Abdallah et al., 2017). Successful *Bacillus* spp. applications for the control of various pathogens of fruits and vegetables during cultivation, handling, transportation and storage have been described, including brown rot in apricots, peach and plums; *Alternaria* rots in litchi, muskmelon and melon; and grey mold disease in apples, pear, citrus and strawberry (Lastochkina et al., 2019).

SA treatment has been shown to delay the post-harvest senescence of various horticultural produce, including goji, mandarins and strawberries, by stimulating the activities and gene expressions of the key proteins (SOD, CAT, APX and POD) associated with redox homeostasis (Zhang et al., 2021). Additionally, the exogenous application of SA was reported to improve the defense of various climacteric fruit or non-climacteric fruits against brown rot, not only by reducing fungi growth but also by enhancing their physical and antioxidant properties as well as the activities of defense-related enzymes (PAL, POD and SOD) (Ezzat et al., 2021). Moreover, SA can delay senescence and the onset of postharvest diseases in horticultural products, making it effective as a natural plant hormone (Haider et al., 2020). SA inhibited the mycelial growth of the pathogenic fungus *Eutypa lata* in a concentration-dependent manner and in either a solid or liquid culture medium (Amborabé et al., 2002). Our results confirmed these effects of SA, which assisted in enhancing the ability of *Bacillus* sp.1–23 to inhibit pathogen growth. Similarly, the composite of cinnamic acid and induced resistance of cinnamic acid-protocatechuic acid-CaCl_2_-NaCl-pullulan (CACP) was used to protect postharvest *H. marmoreus* against *T. harzianum*. The effect mechanism of CACP and SA was similar. The activity of POD, PPO, APX and other oxidoreductases in *H. marmoreus* could also be improved, the content of relative electrical conductivity decreased, and the accumulation of malondialdehyde lessened after CACP treatment, which could also delay the deterioration and aging process in *H. marmoreus* (He et al., 2023). Although the effect against pathogenic microorganisms of these compounds is significant, the possible food safety hazards caused by them cannot be ignored. Some regulatory agencies worldwide have established maximum residue limits (MRLs) and tolerable daily intakes (TDI) for some chemical contaminants (Nascimento et al., 2017). Even in some countries, the use of SA in food is banned (Detpisuttitham et al., 2020).

In this study, *Bacillus* sp.1–23 alone or combined with SA could inhibit the growth of the *Trichoderma* spp. BBP-6 and other saprophytic pathogens to prevent the decay and odor during the storage of white *H. marmoreus*. The effectiveness of this treatment has also been verified in potato tubers during storage. The endophytic *B. subtilis*, both individually or in combination with SA, efficiently alleviated the development of *Phytophthora infestans* and *Fusarium oxysporum* diseases in potato tubers, prolonging their shelf-life and preserving the quality and freshness of their appearance by increasing ascorbic acid content and decreasing pathogen-induced proline accumulation and lipid peroxidation (Lastochkina et al., 2020). However, the safety and safe dosage of SA need to be carefully evaluated before deciding whether it should be used for the harvested *H. marmoreus*.

## 5 Conclusion

Pathogens always infected the harvest white *H. marmoreus*, from which a pathogenic fungus named *Trichoderma* spp. BBP-6 has been successfully isolated. The antagonistic bacteria of *Trichoderma* named *Bacillus* sp. 1–23 was isolated and purified from the environment. *Bacillus* sp. 1–23 produced chitinase and chitosanase enzymes, which could inhibit *Trichoderma* spp. BBP-6 directly. SA could reinforce this inhibitory effect. *Trichoderma* spp. BBP-6 infection was shown to cause severe browning, water and nutrition loss, and quality deterioration in the mushrooms, while *Bacillus* sp. 1–23 and SA separate or combined treatments effectively inhibited the growth of the pathogenic fungus, maintained mushroom nutrients, and reduced the production of MDA, superoxide anion and H_2_O_2_, among others. Moreover, these treatments effectively alleviated the negative effects of the fungal disease by regulating the rate of superoxide anion and H_2_O_2_ content. At the same time, the SOD, CAT, POD, PAL, APX, GR and PPO activities were regulated to a more stable state close to the control group. The findings of this study ascertained the resistance effects of *Bacillus* sp. 1–23 and SA against pathogenic *Trichoderma* spp. BBP-6 in white *H. marmoreus* via the regulation of ROS metabolism. The combination of *Bacillus* sp. 1–23 and SA could be used to mitigate such deterioration and extend the shelf-life of this popular produce. This treatment can be further tried for the postharvest preservation of other agricultural products to confirm the general applicability of this method.

## Data availability statement

The datasets presented in this study can be found in online repositories. The names of the repository/repositories and accession number(s) can be found in the article/supplementary material.

## Author contributions

XY: Conceptualization, Funding acquisition, Project administration, Writing – original draft. TL: Writing – original draft, Conceptualization, Project administration. YL: Data curation, Formal analysis, Methodology, Writing – review & editing. YG: Data curation, Formal analysis, Investigation, Methodology, Writing – review & editing. JL: Data curation, Formal analysis, Investigation, Methodology, Writing – review & editing. CW: Funding acquisition, Investigation, Writing – review & editing. LZ: Funding acquisition, Investigation, Writing – review & editing. XW: Data curation, Formal analysis, Investigation, Methodology, Writing – review & editing. WL: Funding acquisition, Investigation, Writing – review & editing. YS: Data curation, Formal analysis, Investigation, Methodology, Writing – review & editing. FC: Conceptualization, Funding acquisition, Project administration, Writing – original draft. DZ: Conceptualization, Funding acquisition, Investigation, Project administration, Writing – original draft.
